# Future Roadmaps for Precision Medicine Applied to Diabetes: Rising to the Challenge of Heterogeneity

**DOI:** 10.1155/2018/3061620

**Published:** 2018-11-27

**Authors:** P. Bowman, S. E. Flanagan, A. T. Hattersley

**Affiliations:** ^1^University of Exeter Medical School, Exeter, UK; ^2^Exeter NIHR Clinical Research Facility, Royal Devon and Exeter NHS Foundation Trust, Exeter, UK

## Abstract

Precision medicine, the concept that specific treatments can be targeted to groups of individuals with specific genetic, cellular, or molecular features, is a key aspect of modern healthcare, and its use is rapidly expanding. In diabetes, the application of precision medicine has been demonstrated in monogenic disease, where sulphonylureas are used to treat patients with neonatal diabetes due to mutations in ATP-dependent potassium (K_ATP_) channel genes. However, diabetes is highly heterogeneous, both between and within polygenic and monogenic subtypes. Making the correct diagnosis and using the correct treatment from diagnosis can be challenging for clinicians, but it is crucial to prevent long-term morbidity and mortality. To facilitate precision medicine in diabetes, research is needed to develop a better understanding of disease heterogeneity and its impact on potential treatments for specific subtypes. Animal models have been used in diabetes research, but they are not translatable to humans in the majority of cases. Advances in molecular genetics and functional laboratory techniques and availability and sharing of large population data provide exciting opportunities for human studies. This review will map the key elements of future diabetes research in humans and its potential for clinical translation to promote precision medicine in all diabetes subtypes.

## 1. Introduction

Diabetes is a heterogeneous group of metabolic disorders that represents an enormous health burden globally. In 2014, an estimated 422 million adults had diabetes, and the prevalence continues to rise [1, 2]. Complications related to diabetes cause significant morbidity and mortality [1]. At a time when healthcare resources to support an ageing population are limited, it is crucial to develop more effective treatments and make sure that patients receive the treatment appropriate to their condition.

Diabetes is multifactorial and caused by both genetic and environmental factors. Monogenic forms of diabetes (caused by mutations in single genes), including maturity-onset diabetes of the young (MODY) and neonatal diabetes (diagnosed before 6 months of age), are rare, representing ~3.6% of all cases diagnosed under 30 years [3]. Indeed, for most types of diabetes, multiple genes are involved. Type 1 diabetes (T1D) is characterised by insulin deficiency most often resulting from immune-mediated destruction of pancreatic beta (*β*) cells, whilst type 2 diabetes (T2D) results from insulin resistance and *β* cell failure [4]. Also, it is becoming clear that specific subtypes within T1D and T2D have different aetiologies. Correct diagnosis is crucial to allow selection of appropriate therapy, but this can be a challenge for clinicians; even the UK prime minister was misdiagnosed as having T2D and started on the wrong treatment before it became apparent that she had T1D requiring insulin therapy [5]. Indeed, up to 15% of patients with diabetes are misclassified in primary care in England [6]. A recent cross-sectional study showed rates of misclassification are particularly high in those patients with T2D (defined by the presence of significant endogenous insulin secretion more than three years after diagnosis) who are older (>34 years) at diagnosis and who start insulin immediately; they are misclassified as T1D in around half of cases [7]. This experience is not unique to the UK; an 11-year follow-up of an American paediatric diabetes cohort revealed initial misclassification of diabetes in over 20% of individuals [8]. Add to this the heterogeneity within T1D and T2D, and diagnostics and treatment become a major challenge even for the most experienced clinicians.

Precision medicine is the tailoring of treatment to specific molecular or cellular characteristics of groups of patients; this can also be influenced by environmental and lifestyle factors. Precision medicine is rapidly becoming a key concept in many areas of modern clinical practice [9]. Perhaps its most widely recognised application is in oncology, where the specific genetic profile of the tumour can determine the targeted treatment [10, 11]. However, the precision approach has also been applied to other areas of medicine [9], and the field is rapidly developing, largely due to ongoing advances in molecular genetic techniques such as next-generation sequencing (NGS) [12].

There is increasing interest in applying precision medicine to diabetes. In fact, it has already been done in rare monogenic subtypes of the disease, but there are challenges when it comes to applying precision medicine to T1D and T2D [13–15]. One aspect that makes translational research for new targeted treatments particularly challenging in common polygenic subtypes of diabetes is the heterogeneity within these broad disease categories as described above. The first step will be developing an understanding of the often subtle differences in pathophysiology and factors influencing treatment response between individuals with the same “type” of diabetes. This has not been possible using artificial animal models of diabetes. Even though the recent advances in human research methods are more promising than using animals, still some difficulties exist as the lessons learned from monogenic disease are not readily translatable to polygenic diabetes [13].

To facilitate application of a precision medicine approach in diabetes, a comprehensive map of the pathophysiology and treatment targets for each known diabetes subtype is needed, in keeping with the “adverse outcome pathway”-based approach applied to human drug discovery [16]. In this review, we will outline why applying precision medicine to diabetes is unachievable using research findings from traditional animal models and discuss the challenges faced in future translational research in the field.

## 2. Animal Models of Diabetes Are Not Reliably Translatable to Humans

Animals have been used in diabetes research for over a century in an attempt to create models that are relevant to humans [17, 18]. To date, there is no single animal model that accurately represents all aspects of human T1D or T2D. Rodent models have provided some insights into isolated pathways and mechanisms relevant to polygenic diabetes without the time and expense associated with clinical trials and long-term follow-up studies in humans [19]. However, results from these experiments must be interpreted with caution. Most animal models have little relevance to human diabetes; this is exemplified by the problems encountered when attempting to translate animal models of T1D and T2D to humans.

### 2.1. Animal Models in T1D

Early spontaneous rodent models of T1D include the nonobese diabetic (NOD) mouse [20] and the biobreeding (BB) rat [21–23]. In the NOD mouse, insulitis occurs at 3–4 weeks of age and is accompanied by infiltration of islets by CD4 and CD8 lymphocytes, resulting in cytotoxicity and *β* cell destruction with the onset of overt diabetes at around 18 weeks. However, the patterns of insulitis seen in NOD mice are different from those observed in human T1D [24]. In addition, there are significant gender differences in the prevalence of diabetes in NOD mice [25], with females showing the earlier onset and more aggressive disease, likely due to modification of cytokine production and STAT4 gene expression by sex hormones [25]. The gender difference noted in the mice is not apparent in human T1D; this is one of the few circumstances where an autoimmune disease does not occur more frequently in females [26]. BB rats develop diabetes at 8–16 weeks of age and have severe insulin deficiency, but despite not showing the gender differences seen in NOD mice, the rats are lymphopenic [27], which is not a characteristic of T1D in humans. Importantly, in both NOD mice and BB rats, therapeutic interventions for diabetes that have shown promise, e.g., oral insulin and nicotinamide, have not been successful when tried in humans [28, 29]. More recently, the Akita mouse, which has a mutation in the *insulin 2* gene, has been used as a genetically induced model of T1D [30]. These mice show severe insulin deficiency and have a short lifespan; however, caution should be used in extrapolating findings from a monogenic model in a rodent to a more complex polygenic disease in humans, as the pathophysiologies are likely to be different. The lymphocytic choriomeningitis virus (LCMV) rat is a virus-induced model of T1D [31]. LCMV is a rodent-borne virus, but if human infection occurs, there can be neurological sequelae, particularly in the context of congenital infection [32]. However, LCMV has not been linked with diabetes in humans. Indeed, the types of viruses and their precise role in the pathophysiology of T1D in humans are still an active area of research [33]; therefore, the mechanisms of disease are likely to be different to the LCMV-induced rat model.

#### 2.2. Animal Models in T2D

Rodent models of T2D can be categorised into obese and nonobese and are similarly flawed by their inability to fully capture the human phenotype. Nonobese models, generated by selective inbreeding, include the Nagoya-Shibata-Yasuda (NSY) mouse and the Goto-Kakizaki (GK) rat [34, 35]. Similar to the T1D NOD mouse, the NSY mouse shows gender differences in the prevalence of diabetes [25], with a cumulative incidence of diabetes of 98% and 31% at 48 weeks of age in males and females, respectively [34]. This pronounced male excess is not observed in humans with T2D [26]. GK rats have had some utility in the study of diabetes complications and beta cell dysfunction, but limitations include significant heterogeneity between different rodent populations leading to variation in the aetiology of hyperglycaemia, which appears to be mainly due to beta cell dysfunction and/or reduced mass as opposed to insulin resistance [18].

The most widely used models of T2D in animals are the obese models, comprising the monogenic leptin-deficient ob/ob mouse and the leptin receptor-deficient db/db mouse [36, 37]. Both have severe obesity as well as hyperinsulinemic hyperglycaemia [18]. In humans, it is known that monogenic leptin deficiency from either a leptin or a leptin receptor gene mutation is associated with unregulated appetite and very severe obesity [38, 39], but despite this, T2D has not been described to date in these patients. The most severe hyperglycaemia in ob/ob mice occurs at the age of 3–5 months, and the severity decreases thereafter; islet volume in the pancreas is increased, and insulin secretion is maintained [40]. This process does not reflect the *β* cell failure seen in human T2D. In db/db mice, ketosis occurs at a few months of age, and they do not live long (only 8–10 months) [41]. Again, this does not reflect the natural history of T2D in humans. For full detailed reviews of animal models in diabetes, see King, *British Journal of Pharmacology* [18].

#### 2.3. Animal Models in Monogenic Diabetes

Rodent models of monogenic diabetes have tended to follow on from the discoveries of single gene aetiologies in humans. They have had some utility in providing support for hypotheses relating to mechanism and expression patterns for specific genes, particularly in MODY caused by mutations in the transcription factors hepatocyte nuclear factor 1 alpha (*HNF-1A*) [42, 43] and hepatocyte nuclear factor 1 beta (*HNF-1B*) [44] and in neonatal diabetes due to *KCNJ11* mutations [45–47]. However, the phenotype of the monogenic mouse, both in relation to diabetes and extrapancreatic features, is not always consistent with what is observed in humans [48, 49]. In addition, the natural history of disease may differ; for example, humans with glucokinase MODY do not have renal complications in the long term which contrasts with the proteinuria and structural kidney changes observed in a liver-specific hemizygous glucokinase knockout mouse model [50, 51]. These issues limit translatability of such animal models to monogenic diabetes in humans.

## 3. Human Research Is Needed to Address the Questions That Cannot Be Answered Using Animals

The fundamental differences in the natural history of T1D and T2D in animal models and humans make it impossible to interrogate these broad disease categories at an individual or indeed subgroup level using rodents. Monogenic diabetes rodent models bear a slightly closer resemblance to their human equivalents, but clinical translation remains limited. As research in animals does not provide the insights into the heterogeneity of diabetes that are required for therapeutic advances in the field, new approaches, focusing on research in humans, are needed (Table 1).

### 3.1. Advances in Human Molecular Genetics Have Driven Treatment Change and Improved Clinical Care in Monogenic Diabetes

We have outlined the significant limitations of using a monogenic disease in animals to model a disease that is polygenic in humans. However, one key question is whether we can learn lessons from monogenic diabetes in humans that are generalisable to polygenic forms of diabetes.

Advances in human genetics have revolutionised monogenic diabetes research and clinical care for affected families by accelerating gene discovery and allowing better treatments to be developed for some subtypes. Historically, single candidate genes for a disease in question were screened using Sanger sequencing. This is an accurate method of sequencing, but the analysis is relatively slow and expensive as single genes need to be analysed sequentially in sections (by exon). Sanger sequencing of specific genes is therefore not ideal for disorders where there is significant overlap in phenotype both within and between different genetic aetiologies or where the genetic cause is not yet known. Next-generation sequencing is a relatively new technique that allows sequencing of many genes all at once, at a similar cost to sequencing just a few genes by the traditional Sanger method [12]. This is highly advantageous in monogenic diabetes, where an early and rapid genetic diagnosis is crucial for two reasons. Firstly, there are treatments that are available for specific types of diabetes but not for others. For example, maturity-onset diabetes of the young due to *HNF1A/4A* mutations can be treated with low-dose sulphonylureas; neonatal diabetes due to potassium channel gene mutations can be treated with high-dose sulphonylureas, whereas mild fasting hyperglycaemia due to glucokinase mutations does not require pharmacological treatment [13].

Secondly, early identification of diabetes caused by a single gene allows early prediction of other (extrapancreatic) clinical features associated with that specific gene, facilitating provision of necessary support and interventions soon after diagnosis; in the case of neonatal diabetes, this would be in the first six months of life. This contrasts with previous approaches where clinicians would have to wait for the patient to develop extrapancreatic features before determining which genes to sequence [52]. In neonatal diabetes, a genetic diagnosis can now be made in 80% of cases [52], because all babies who present with diabetes in the first 6 months of life can have a panel of known disease-causing genes sequenced rapidly and accurately using the NGS method.

### 3.2. Humans with *KCNJ11* Mutations Represent the Best Example of Precision Medicine in Diabetes

A good example of precision medicine in monogenic diabetes is the treatment of *KCNJ11* neonatal diabetes with sulphonylureas [53]. *KCNJ11* encodes the Kir6.2 subunit of the pancreatic ATP-dependent potassium (K_ATP_) channel; it is present in *β* cells and links blood glucose to insulin secretion. In 2004, the sequencing of *KCNJ11* in human subjects established mutations in this gene as a cause of permanent neonatal diabetes (PNDM) [54]. PNDM affects ≈1/100,000 live births [55] and is defined as diabetes diagnosed within the first 6 months of life. To date, there have been 24 genetic causes of neonatal diabetes identified [52, 56, 57], and *KCNJ11* mutations are the commonest cause accounting for around one-third of all cases [52].

*KCNJ11* mutations result in diabetes by rendering the K_ATP_ channel unresponsive to metabolically generated ATP. Affected babies are clinically very sick and show insulin deficiency, with almost 80% presenting in diabetic ketoacidosis (DKA) [58]. Until pathogenic variants in the *KCNJ11* gene were discovered, these children were thought to have T1D and were treated with insulin injections [54]. Physiological experiments in affected individuals highlighted the possibility that sulphonylureas, used in T2D to bind and close the K_ATP_ channel, could be used as a targeted treatment option in *KCNJ11* PNDM. This was confirmed in 2006 when the first large cohort study showed that 90% of patients were able to switch from insulin injections onto oral sulphonylureas with improvements in glycaemic control and less glycaemic variability [53, 59]. Inability to switch, although uncommon, is associated with specific genotypes and long duration of diabetes before attempting to change treatment [60, 61]. In those who switch successfully, the excellent initial glycaemic response is maintained over at least 5 years and is not associated with any increase in hypoglycaemia rates [62–64].

The repurposing of an existing oral diabetes therapy that resulted in near normalisation of blood glucose for the great majority of affected individuals with *KCNJ11* PNDM was life-changing for patients and their families, and human research was crucial for this discovery. Indeed, without the gene discovery and the clinical trial of targeted therapy in humans, people with *KCNJ11* PNDM would have remained on a treatment that was not very efficient and that allowed only suboptimal glycaemic control, leading to increased risk of long-term diabetes complications.

### 3.3. Neurological Features in *KCNJ11* PNDM Reflect Expression of the *KCNJ11* Gene in the Brain and Vary according to Genotype

Initial reports of *KCNJ11* PNDM showed that ≈20% of affected individuals exhibited overt and severe neurological features in addition to their diabetes; this was named DEND syndrome (developmental delay, epilepsy, and neonatal diabetes) or intermediate DEND (iDEND) if epilepsy was not evident in the first 12 months of life. The clinical phenotype was found to be related to the genotype, with more severe clinical features generally being associated with the more functionally severe mutations [49, 65]. For example, early studies reported developmental delay/intellectual disability (often severe), motor problems, and/or epilepsy in ≥80% of patients with the V59M mutation, in contrast to the R201H mutation where diabetes without neurological features was reported in >95% cases [54, 59, 65–76].

The presence of neurological features in this type of diabetes is due to expression of *KCNJ11* in K_ATP_ channels in several brain regions as well as the pancreas [77]. Recent research has shown that in addition to the classical DEND syndrome, patients can have a range of other specific features. Neurodevelopmental disorders such as autism and ADHD are more commonly associated with specific mutations like V59M [78, 79]. Furthermore, neuropsychological impairments affecting executive function, attention, praxis, working memory, vocabulary, and visuomotor performance have been identified [78, 80–82]. Interestingly, subtle abnormalities are also observed in patients with mutations previously thought to cause only diabetes without neurological features. One large cohort study of patients without overt neurological features reported attention deficits in all patients and dyspraxia (developmental coordination disorder) in 80% [80].

Performing this detailed phenotyping in humans has provided clinical insights that would not have been possible using nonhuman research methods. For example, selective expression of the V59M mutation in the rodent brain gives rise to a model of DEND syndrome which shares characteristics with the human neurological phenotype [46]. However, there are also notable differences, e.g., the mice show reduced anxiety behaviour whereas humans show more anxiety [47, 78]. In addition, the milder neurological phenotypes associated with other mutations in the same gene have not been explored in rodent models, and subtle cognitive deficits would be very difficult to assess in animals in the same way as they can be assessed in humans.

### 3.4. Impact of Sulphonylureas on the Neurological Phenotype in *KCNJ11* PNDM and Generating Mechanistic Hypotheses from the Rodent Model

In addition to achieving excellent metabolic control, an exciting aspect of switching patients with *KCNJ11* mutations from insulin to sulphonylureas, which was initially described in clinical case reports and neuroimaging studies, is an improvement in the neurological features [75, 83–86]. This was recently confirmed by a prospective study which showed partial improvement in some of the neurological features in the first year after switching to sulphonylureas [87]. It has been suggested that the neurological response may be better the earlier in life the sulphonylureas are started [82], due to increased neuroplasticity in younger children, but further studies are needed to address this issue.

Another possible reason for the incomplete CNS response to sulphonylurea treatment in people with *KCNJ11* PNDM is that therapeutic concentrations of sulphonylurea are not achieved in the human CSF. In rats, active transport of glibenclamide out of the brain across the blood–brain barrier (BBB) has been demonstrated [88]. Therefore, high concentrations of glibenclamide, as seen in the blood, are not achieved in the brain. This concept has led to the use of higher doses of sulphonylureas in individuals with neurological features, with improvements reported by patients at doses of around 1 mg/kg/day glibenclamide. These higher doses appear to be safe with no increase in rates of hypoglycaemia [89]. However, given the issues around translation of animal models outlined above and the structural differences between the rodent and human brain [90], it will be important to confirm in future human studies if and how glibenclamide and other sulphonylureas act in the human CNS. This might include direct *in vivo* measurement of sulphonylurea concentrations in human cerebrospinal fluid (CSF) or the use of *in vitro* experiments with BBB models [91] which may provide a potential way of investigating this question without the risks of invasive procedures in patients.

### 3.5. Lessons Learned from *KCNJ11* PNDM Are Not Directly Applicable to All Neonatal Diabetes or to Polygenic Forms of Diabetes

**Figure 1:**
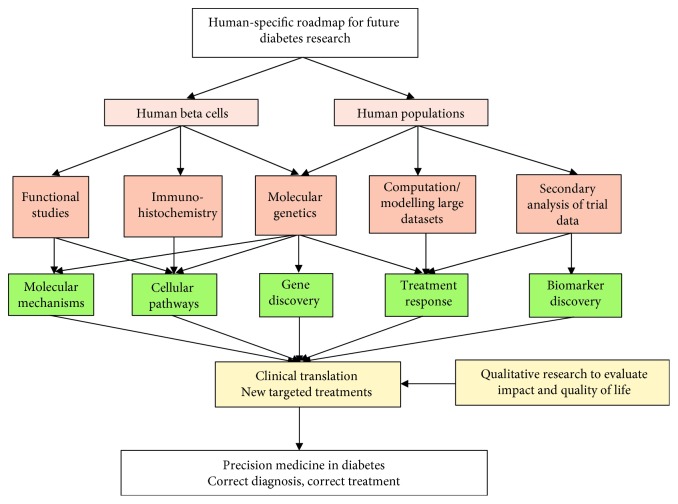
Human-specific research methods (orange boxes) can be applied to key areas (green boxes) relevant to diabetes pathophysiology, leading to development of new targeted treatments.

*KCNJ11* PNDM is a good example of how human molecular genetics has driven the application of precision medicine in diabetes. However, *KCNJ11* mutations are only one of the causes of neonatal diabetes, and findings in one subtype are not generalisable to all, although the general concept of using molecular genetics to determine aetiology and treatment can be applied more widely (Figure 1). Other subtypes of neonatal diabetes are caused by mutations in a variety of genes; all share the clinical characteristic of diabetes in the first 6 months of life, but there are significant phenotypic differences between them. For example, people with neonatal diabetes due to insulin (*INS)* gene mutations (which account for around 10% of cases of neonatal diabetes) do not have any specific neurological phenotype [92], whereas CNS features comprise a large part of the phenotype in *KCNJ11* PNDM. Individuals with other syndromic forms of neonatal diabetes have neurocognitive impairments in addition to other multisystem features, e.g., *GATA6* mutations cause cardiac defects, pancreatic exocrine insufficiency, gut abnormalities, and hypothyroidism/hypopituitarism [93].

In addition to phenotypic differences, differing genetic aetiologies also mean that different treatment approaches are needed. Heterozygous dominant negative *INS* mutations cause abnormal preproinsulin and proinsulin structures to be produced. This causes ER stress in the beta cell resulting in cell death and absolute insulin deficiency [52] which requires permanent insulin treatment [94], in stark contrast to the sulphonylurea sensitivity of patients with *KCNJ11* mutations [53]. Even within *KCNJ11* neonatal diabetes, there is heterogeneity amongst patients with the same mutation in terms of phenotype and treatment responses, as described above. This heterogeneity is true for all subtypes of diabetes, including the common polygenic forms (T1D and T2D); however, it provides an opportunity to define discrete subgroups in a precise manner, with significant implications for new drug discovery and repurposing of existing treatments.

## 4. A Human-Specific Roadmap for Future Diabetes Research

We have established that findings obtained with animal models are not efficiently translated into humans, and it is impossible to generalise research findings from one subtype of human diabetes to another. Therefore, alternative approaches are needed to drive advances in diabetes research that are clinically translatable. A range of rapidly evolving methods can be applied to human cells and human populations to enhance understanding in key areas, facilitating development of new targeted treatments between and within all subtypes of diabetes and allowing application of precision medicine (Figure 1).

### 4.1. The Impact of Molecular Genetics in T1D and T2D: Aetiology and Correct Diagnosis

Before we can develop effective new therapies in diabetes, we must identify and understand aetiological pathways that can provide targets for treatment. One of the ways in which human molecular genetics has enhanced our understanding of the pathophysiology of polygenic diabetes is through genome-wide association (GWA) studies [95, 96]. These have been made possible by development of high throughput genotyping technologies such as NGS, increased availability of large cohorts of individuals with the disease in question and control population data with which to compare them (see below), and better understanding of sequence pattern variation [96]. Over 100 T2D susceptibility loci have been identified to date, and there is now much focus on determining the function of associated genes and the pathways in which they play a role [97]. However, interpretation of the function of associated genetic variants is challenging as it is frequently difficult to prove a causal link between the variant and the disease [97]. In addition, effect sizes of causal variants in T2D are small [96], making it extremely difficult to develop specific therapies targeted at a single gene or pathway, as has been described above for monogenic diabetes. For these reasons, clinical translation of GWAS findings has been limited to date. In the future, as whole genome sequencing becomes less costly, it is likely that larger populations will be screened which may assist the discovery of new variants or help explain existing associations and how they relate to T2D risk. Further advances in functional experimental techniques may enhance our ability to move from associations to causal relationships. T2D GWAS will therefore be an important tool in terms of biological insights, drug targets, and disease prediction (Figure 1).

Despite the complexities of functional interpretation of genetic risk variants in polygenic diabetes, they can be useful in assisting diagnosis, which is fundamental for selecting the correct treatment. In T1D, a genetic risk score (T1D GRS) based on 30 T1D-associated risk variants each weighted according to individual risk contribution has been developed; this score or derivatives comprising even fewer SNPs can reliably differentiate T1D from T2D and T1D from monogenic diabetes [98, 99]. The T1D GRS is now being used in both research and clinical contexts. This has significant implications in terms of making the correct clinical diagnosis early and starting the correct treatment, as well as ensuring phenotypic purity in research cohorts in T1D. As it is a relatively low-cost investigation, its use is likely to become more widespread in the future, and there is potential for similar methodology to be applied to other polygenic diseases.

Finally, genes involved in monogenic diabetes may also be implicated in polygenic disease, for example, activating mutations in *KCNJ11* causes neonatal diabetes whilst the common E23K variant in *KCNJ11* has been associated with T2D susceptibility [54, 100]. Therefore, monogenic diabetes has utility in identifying potential mechanisms that contribute to polygenic diabetes risk [101]. However, the complex inheritance patterns, multifactorial aetiologies, and small effect sizes of genetic risk variants in polygenic diabetes give rise to very heterogeneous populations of patients, and multiple complementary approaches are required to unpick this.

### 4.2. Availability of Large-Population-Based Data Sets and Sharing of Data Can Provide New Insights into Polygenic Diabetes

Historically, one of the drawbacks of research in humans has been the inability to power studies adequately due to lack of availability of cohorts of patients with a specific disease or aspects of a disease of interest. This is particularly problematic in genetics studies, where large populations are required to identify risk variants with relatively small effect sizes in polygenic diseases like T1D and T2D. In recent years, the problem has been mitigated by the availability of increasing numbers of large-population research cohorts, such as UK Biobank [102]. UK Biobank contains anonymized health data, including genetic and clinical information, on over 500,000 volunteers which are available for approved researchers to use. Application of rapidly advancing bioinformatics techniques to these population-based data sets represents an exciting opportunity to gain novel insights. Indeed, one recent publication using UK Biobank and applying the T1D GRS outlined above [99] provided new insights into T1D, by demonstrating persistence of T1D risk beyond the age of 30 thereby highlighting the need for clinicians to continue to consider this diagnosis in adults [103].

Another means of acquiring data from large populations is data sharing from large-scale clinical trials, which is now actively encouraged and endorsed by many trial sponsors and influential bodies [104]. Full individual participant data for many trials can be requested and accessed by researchers via websites such as Clinical Study Data Request [105]. Secondary analysis and statistical modelling of trial data allow evaluation of outcomes in subgroups of patients based on clinical characteristics, the presence of specific biomarkers, or genotype. These population-based methods can facilitate an alternative approach to precision medicine in polygenic forms of diabetes, such as T2D, whereby clinical features and biomarkers are used to stratify patients into specific treatment groups [13]. An excellent example of this is using clinical features to stratify patients with type 2 diabetes when deciding which second-line glucose-lowering therapy to use [106]. Therefore, future clinical research on diabetes will rely heavily on shared human population data.

The concept of large-scale data sharing is also applicable to genetic data. NGS technologies have reduced the cost of genetic testing by a factor of between 100 and 200 in the last 5 years. As genetic testing continues to decrease in price and analysis methods improve, sequencing particularly at the level of exome or whole genome will become more accessible to larger numbers of individuals. This will generate vast quantities of genetic data requiring accurate interpretation, which can be a major challenge. However, in recent years, databases generated from data sharing containing genetic variants from human populations (e.g., the Genome Aggregation Database (gnomAD), Exome Aggregation Consortium (ExAC), and dbSNP) and human disease (e.g., the Human Gene Mutation Database (HGMD) and ClinVar) have revolutionised the ability of clinical scientists to interpret variants and their likely pathogenicity. Further initiatives such as the 100,000 Genomes Project seek to not only provide clinical diagnoses for people with rare genetic conditions but also generate a large population sample of genomic data that will be invaluable for researchers in the future as the patients' health records and outcome data can be linked to their genetic data [107].

Indeed, the concept of integrating research with clinical practice has evolved substantially in recent years, particularly where there is availability of electronic health records (EHR). Primary care is particularly well placed to apply this because many practices have moved to an EHR approach. In the UK, Clinical Practice Research Datalink (CPRD) is a well-established source of anonymized clinical information from general practice (GP) records that can be utilised for research; it has resulted in over 1800 publications to date [108]. Most patients with diabetes are followed up clinically by their GP; therefore, this is a key opportunity for research in the field. Indeed, it has been shown that diabetes and its treatment are two of the main topics of research being generated from primary care databases in the UK [109]. However, there are several legal and ethical issues relating to data sharing and storage that have hindered the use of EHR in many healthcare settings; this is particularly pertinent when it comes to linking genomic data with personally identifiable data [110, 111]. To make the most of the opportunities afforded by EHR in the future, robust policies addressing confidentiality and security of information should be developed by key regulatory authorities [112].

A caveat of the clinical and genomic data repositories that are currently available is the paucity of ethnic diversity in the populations studied leading to underrepresentation of non-European groups [113]. The ever-increasing number of individuals contributing data to such repositories bodes well for improved stratification by ethnicity in the future, but in the meantime, caution should be used when attempting to generalise findings to minority populations.

### 4.3. Availability of Human Islets for Research and New Experimental Techniques Provide Insights into Pathways Involved in Diabetes Pathophysiology

Modern immunohistochemical and imaging techniques and availability of collections of specific human tissues for research can greatly enhance our understanding of the pathophysiology of diabetes. A recent study of pancreas sections obtained at postmortem from a UK cohort of patients with T1D provided exciting mechanistic insights, demonstrating a different insulitic profile in patients diagnosed under 7 years versus those diagnosed over 13 years [114]. In addition, the latter group retained ~40% of insulin containing islets at diagnosis, which implies *β* cell dysfunction as opposed to loss may be important. This work and ongoing research in the field will have important implications for patient stratification in T1D immunotherapy trials and in the development of targeted treatments for specific patient groups.

Research in human islets harvested from cadaveric donors has also advanced knowledge relating to cellular and molecular pathways relevant to T2D. Recent advances in genetic techniques have facilitated identification of many T2D susceptibility genes and allowed genetic data to be combined with functional data to map pathways and define mechanisms associated with human islet dysfunction, including key regulatory networks [115, 116]. These approaches have great potential to further enhance our understanding of polygenic forms of diabetes and gene-environment interactions and in combination with findings from large population studies, to guide development of new therapeutic interventions.

### 4.4. Precision Medicine Must Also Encompass Patient Preference and Impact on Quality of Life

Another area of precision medicine where human studies are essential is exploring the influence of psychosocial factors on patient outcomes. Quality of life measures are frequently used in evaluating cost-effectiveness of medical interventions [117]. Development of targeted treatments for specific subtypes of diabetes should therefore include research that evaluates patients' perceptions of these treatments and impact on quality of life. Even when the biological efficacy of new treatments has been proven, the willingness of patients to accept them will be variable and influenced by psychological factors. For example, treatment change from insulin injections to oral sulphonylureas had a hugely positive impact on many families affected by *KCNJ11* neonatal diabetes. They experienced improved quality of life, more freedom, and reduced levels of psychological distress as a result of better glycaemic control, less glycaemic variability, and reduced need for hypervigilance of parents towards their affected children [118–120]. However, for a few adults with *KCNJ11* mutations who had been assumed to have T1D all of their lives, there was initial uncertainty about the implications of a genetic diagnosis as it could result in a loss of the insulin injections on which they had always been dependent [118, 119]. These individuals viewed insulin very much as part of their identity, and loss of this identity required significant adjustment [121].

In addition, mental illness is a significant problem in individuals with chronic physical health conditions. The incidence and prevalence of depression are increased in people with diabetes [122], which will have implications for adherence, response, and attitudes to new treatments. Severe mental illnesses such as schizophrenia and bipolar disorder are associated with a 2–3 fold increase in diabetes prevalence, and this is only partly explained by the adverse metabolic effects of antipsychotic treatment [123]. Patient stratification using only biomarkers or genetic risk variants for diabetes does not take account of psychological influences and psychiatric comorbidity. Future models for precision approaches in diabetes should incorporate these ideas; this will be challenging but could be facilitated by integration of qualitative methods into biological studies and interdisciplinary collaboration.

## 5. Human-Specific Research Can Enhance Understanding of Heterogeneity and Is the First Step towards Precision Medicine across All Subtypes of Diabetes

In diabetes, the correct diagnosis is essential to ensure the correct treatment is given. However, both diagnostics and therapeutics continue to represent significant challenges to diabetologists. Heterogeneity between and within subtypes of diabetes is becoming increasingly recognised and only serves to make the task more difficult. To enable a precision medicine approach in diabetes, we need to significantly enhance our understanding of this heterogeneity.

Animals have been used historically to model diabetes in humans, but their utility is limited largely because the emphasis in humans is on specific treatments for specific diabetes subtypes. The animal models used have fundamental genetic and phenotypic differences to diabetes in humans and cannot reflect the diversity of subtypes. This is exemplified by the lack of effective translation of treatments developed in animal models into humans. Therapeutic advances in diabetes therefore require alternative human-specific research methods.

Monogenic diabetes is an excellent example of the application of precision medicine. In particular, the treatment of *KCNJ11* neonatal diabetes with sulphonylureas represents the best precision approach in diabetes and illustrates how advances in human molecular genetic techniques have facilitated major discoveries, with huge implications for patient care. However, it also illustrates how specific targeted treatment for one subtype within a broader category (in this case, neonatal diabetes) cannot be generalised to all subtypes. In polygenic diabetes such as T1D and T2D, genetics can help by providing information about risk variants, but effect sizes are small. The situation is particularly complex given that within T1D and T2D there is significant heterogeneity between groups of individuals, whether they are defined by clinical characteristics or response to treatment.

In summary, the road ahead in diabetes research is exciting but complex. A combined approach that uses advanced molecular genetic techniques, pathway-focused research in human islets, computational methods in large population cohorts and trial data, qualitative research, and other techniques yet to be developed may help to unpick the differences between diabetes subtypes. This will be the first step towards understanding and rising to the challenge of heterogeneity in diabetes, to facilitate precision medicine and improved clinical care.

## Figures and Tables

**Table 1:** Opportunities and limitations in diabetes research.

Models in diabetes research		Utility	Limitations	Facilitators	Future potential
Human	Populations	(i) GWAS for risk variants in polygenic disease and new gene discovery studies for monogenic disease (ii) Risk and treatment stratification using biomarkers and clinical features (iii) Clinical trials of new/repurposed treatments	(i) Large-scale bioinformatics support and data management/storage required with cost implications (ii) Ethical implications of use and long-term storage of genetic data (iii) Functional and clinical interpretation of genetic data is challenging particularly for vast quantities	(i) High throughput genomic sequencing techniques, e.g., NGS (ii) Data sharing via human gene/disease/clinical databases, clinical trial data access (iii) Integration of research into clinical practice, e.g., 100,000 Genomes Project (iv) Electronic health records	+++
	Beta cells	(i) Mapping pathways and regulatory networks in combination with molecular genetic data (ii) Determining the role of immunological/environmental factors	(i) Difficult to obtain large numbers of specimens from cadaveric donors (ii) Does not capture multisystem physiology and so may not be fully translatable to the whole organism	(i) High throughput genomic sequencing techniques, e.g., NGS (ii) Improved interpretation of GWAS findings (iii) Advances in laboratory techniques	++
Animal	Induced	(i) Can provide some supporting evidence of disease causality or association for genetic/environmental factor(s) being studied	(i) Differences in aetiology and natural history of disease between animals and humans limit clinical translation/utility (ii) Not useful for testing therapeutic interventions as differences in animal and human responses	(i) Advances in molecular genetic techniques including genetic manipulation	−/+
	Spontaneous	(i) May help generate hypotheses about factors involved in disease aetiology/pathophysiology			−/+

NGS = next-generation sequencing; GWAS = genome-wide association study; +++ = excellent potential for future advances; ++ = good potential; + = possible potential; − = limited potential.
